# No high level evidence to support the use of oral H1 antihistamines as monotherapy for eczema: a summary of a Cochrane systematic review

**DOI:** 10.1186/2046-4053-3-25

**Published:** 2014-03-13

**Authors:** Esther J van Zuuren, Christian J Apfelbacher, Zbys Fedorowicz, Aldrin Jupiter, Uwe Matterne, Elke Weisshaar

**Affiliations:** 1 Department of Dermatology, B1-Q, Leiden University Medical Centre, Leiden, the Netherlands; 2 Medical Sociology, Department of Epidemiology and Preventive Medicine, University of Regensburg, Regensburg, Germany; 3 Bahrain Branch, The Cochrane Collaboration, Awali, Bahrain; 4 Department of Clinical Social Medicine, Occupational and Environmental Dermatology, Heidelberg University Hospital, Heidelberg, Germany

**Keywords:** Cochrane review, eczema, H1 antihistamines, evidence based medicine

## Abstract

**Background:**

The most important symptom as well as one of the major diagnostic criteria for eczema is itch. Although oral antihistamines continue to be prescribed for people with eczema, it is unclear if they are effective and safe in relieving itch and skin lesions. We sought to evaluate the available evidence on effectiveness of oral antihistamines (H1 antagonists) as monotherapy in children and adults with eczema.

**Methods:**

Searches included 10 databases and trial registers as well as conference proceedings (January 2014). Randomised controlled trials that assessed the effects of oral H1 antihistamines as monotherapy in children and adults with eczema were included.

**Results:**

Our searches retrieved 757 references, but no randomised controlled trial met our inclusion criteria. Most studies allowed concomitant treatments, making the assessment of the individual effects of oral H1 antihistamines impossible.

**Conclusions:**

There is currently no high-level evidence to support or refute the efficacy or safety of oral H1 antihistamines used as monotherapy for eczema. A further review of studies that assesses the effects of oral H1 antihistamines as ‘add-on’ therapy together with concomitant treatments is warranted to determine the beneficial effects of this group of medications in the treatment of eczema.

## Background

Eczema is a common chronic inflammatory skin disease with an estimated prevalence ranging from 2% to approximately 20% worldwide [1,2]. The concurrent use of terms such as “eczema”, “atopic eczema”, “atopic dermatitis”, “atopic eczema/dermatitis syndrome” and “neurodermatitis” has led to confusion and inconsistency in their application. Therefore, the nomenclature review committee of the World Allergy Organization has proposed the use of "eczema" as a unifying term, and this term is also used throughout the text of this review [3]. The majority of cases occur before the age of 5 years and, although eczema often resolves during childhood or adolescence, relapses do occur and the disease can persist into adult life [4]. Itch is the most important symptom with a major impact on health-related quality of life [5]. It is one of the major diagnostic criteria for eczema, and without itch the diagnosis of eczema cannot be confirmed [4]. A wide range of options are available for the treatment of eczema and antihistamines are often prescribed to relieve the associated itch [4]. Although the exact role of histamine in itch still needs to be elucidated and it remains unclear if oral antihistamines are effective in the management of eczema, they continue to be prescribed by physicians.

This is a summary of a Cochrane systematic review that was conducted to assess the effects of oral antihistamines (H1 antagonists) as monotherapy in children and adults with eczema [4].

## Methods

A systematic review of randomised controlled trials (RCTs) according to a prespecified protocol [4].

### Search strategies

We searched for relevant studies in 10 electronic databases and trial registers up to January 2014 (see Table 1). The bibliographies of the excluded studies were examined for further references to potentially eligible RCTs (EJvZ) and two review authors searched for ongoing and unpublished trials in January 2014 (EJvZ and UM). Abstracts from the International Research Workshops on eczema and itch, the conference proceedings of the European Academy of Dermatology and Venereology, and of the European Academy of Allergy and Clinical Immunology from 2000 to 2011 were screened, in order to identify further potentially relevant RCTs (EJvZ, UM, and EW). No language restrictions were imposed. Four review authors independently assessed the titles and abstracts for eligible RCTs (CJA, EJvZ, ZF and AJ).

**Table 1:** Electronic databases and trial registers searched

**Electronic databases**	**Trial registers**
The Cochrane Skin Group Specialised Register	The metaRegister of Controlled Trials http://www.controlled-trials.com
The Cochrane Central Register of Controlled Trials MEDLINE	The US National Institutes of Health Ongoing Trials Register (http://www.clinicaltrials.gov)
EMBASE	The Australian and New Zealand Clinical Trials Registry (http://www.anzctr.org.au)
LILACS	The World Health Organization International Clinical Trials Registry platform http://www.who.int/trialsearch
	The Ongoing Skin Trials Register on http://www.nottingham.ac.uk/ongoingskintrials

### Inclusion/exclusion criteria

Inclusion criteria were RCTs in children and adults with a clinical diagnosis of eczema identified as ‘atopic eczema’ or ‘eczema’ that compared oral antihistamines (H1-antagonists) of all classes (sedating, non-sedating) as monotherapy versus placebo or alternative treatment (for example, topical corticosteroids or topical immunomodulators). We excluded studies investigating the following interventions and comparisons: one antihistamine versus another antihistamine; and topical antihistamines and H1 antihistamines as 'add-on' therapy other than emollients/moisturisers.

### Outcome measures

Our two primary outcomes were (i) reduction of subjectively perceived itch, as determined by validated rating scales (for example, visual analogue scales) and (ii) global improvement in eczema, as measured by reduction in an eczema severity score such as SCORAD [6] or any other scoring scale. Secondary outcomes were percentage of participants reporting adverse effects/adverse events, and improvement in quality of life measures using any generic or disease-specific instrument.

### Data extraction and synthesis

No studies met our inclusion criteria. If any eligible studies are identified for future updates of the Cochrane review, the following methods of data extraction and synthesis will apply. Study details will be extracted and summarised using a structured data extraction form by two review authors. Risk of bias will be independently assessed for each included study using the Cochrane Collaboration’s domain-based evaluation tool as described in Chapter 8, Section 8.5, in the *Cochrane Handbook for Systematic Reviews of Interventions*[7]. For the statistical analysis, dichotomous outcomes will be presented as relative risk ratios and continuous outcomes as mean differences if the same measurement scales have been used across trials. Standardised mean differences will be used to express results for continuous outcomes if different, but comparable, measurement scales have been used across trials. All outcomes data will be reported with their associated 95% confidence intervals. We will perform a meta-analysis for studies with similar types of interventions to calculate a weighted treatment effect across the trials, using a random effects model. Clinical heterogeneity will be assessed by examining the characteristics of the studies, in terms of the similarity between the types of participants, the interventions and the corresponding outcomes. Statistical heterogeneity will be assessed using a Chi^2^ test and the *I*^*2*^ statistic. We will report heterogeneity as moderate to high for *I*^*2*^ values between 30% and 60% and as substantial between 50% and 90% [7].

## Results

**Figure 1:**
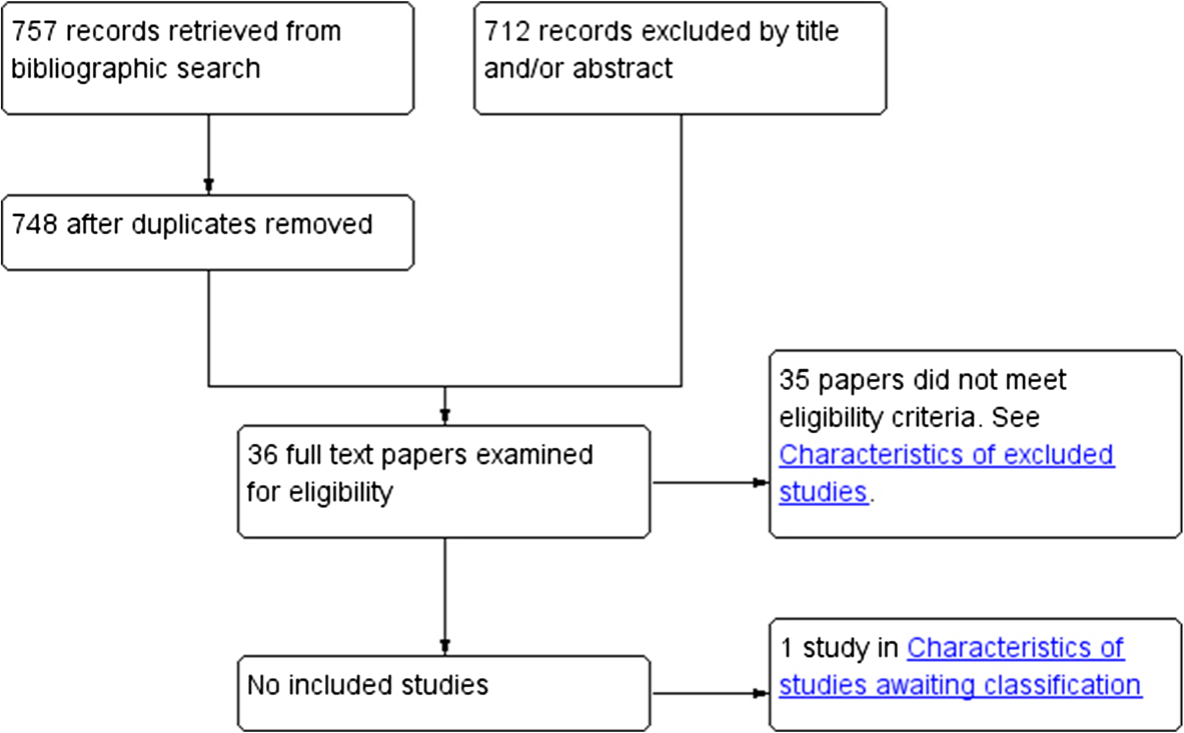
Search results.

From the searches we identified 757 studies which, after removal of the duplicates, provided a total of 748 citations (see Figure 1). After examination of the titles and abstracts a further 712 ineligible studies were eliminated. Full text copies of the remaining 36 studies were obtained and these were assessed independently for eligibility by four authors (CJA, EJvZ, ZF and AJ). No ongoing trials matching our inclusion criteria were identified nor were any eligible studies found after screening the abstracts of all of the potentially relevant conference proceedings. Finally, 35 out of the 36 remaining studies were excluded and one study is pending assessment as we await a reply from the principal investigator about missing and incomplete trial details [8]. The majority (18) of these excluded studies allowed the use of certain concomitant treatments which were pre-specified as exclusion criteria for study eligibility in this systematic review. A further eight studies were excluded as these compared one antihistamine with another antihistamine, which was another pre-specified exclusion criterion. We excluded the remainder (9) for other reasons (for example, non-RCTs or other skin diseases).

## Discussion

Unfortunately no RCTs could be included in our Cochrane review (see Table 2). Most of the studies retrieved in our searches had examined the effect of oral H1 antihistamines as 'add-on' therapy to other topical or systemic treatment, and consequently did not permit reliable assessments to be made of the individual effects of H1 antihistamines. Topical and/or systemic treatments used concomitantly should be regarded as modifiers of the individual effect of the H1 antihistamines and can potentially confound any assessment of their efficacy.

**Table 2:** **Oral H1 antihistamines compared to placebo or alternative treatment for eczema: summary of findings**[9]

	Illustrative comparative risks* (95% CI)					
	**Assumed risk**	**Corresponding risk**				
**Outcomes**	**Placebo or alternative treatment**	**Oral H1 antihistamines**	**Relative effect (95% CI)**	**No of participants (studies)**	**Quality of the evidence (GRADE)**	**Comments**
Reduction of subjectively perceived itch, as determined by validated rating scales	-	-	Not estimable	0 (0)	No evidence from RCTs that met our inclusion criteria^1^	No RCT could be included
Global improvement in eczema, as measured by reduction in an eczema severity score such as SCORAD	-	-	Not estimable	0 (0)	No evidence from RCTs that met our inclusion criteria^1^	No RCT could be included
Percentage of participants reporting adverse effects/adverse events	-	-	Not estimable	0 (0)	No evidence from RCTs that met our inclusion criteria^1^	No RCT could be included
Improvement in quality of life measures	-	-	Not estimable	0 (0)	No evidence from RCTs that met our inclusion criteria^1^	No RCT could be included

Patient or population: patients with eczema. Intervention: oral H1 antihistamines. Comparison: placebo or alternative treatment (excluding: one antihistamine versus another antihistamine; topical antihistamines; H1 antihistamines as 'add-on' therapy; and studies using any concomitant therapy other than emollients/moisturizers).

*The basis for the assumed risk (for example, the median control group risk across studies) is provided in footnotes. The corresponding risk (and its 95% CI) is based on the assumed risk in the comparison group and the relative effect of the intervention (and its 95% CI).

GRADE Working Group grades of evidence: high quality, further research is very unlikely to change our confidence in the estimate of effect; moderate quality, further research is likely to have an important impact on our confidence in the estimate of effect and may change the estimate; low quality, further research is very likely to have an important impact on our confidence in the estimate of effect and is likely to change the estimate; very low quality, we are very uncertain about the estimate.

^1^There might be some evidence coming from studies that allowed concomitant therapies for eczema, but that would not address the singular effect of oral antihistamines on eczema.

Although oral H1 antihistamines (especially of the sedating type) are widely prescribed for the relief of itch, our systematic review indicates that there is currently a lack of evidence to support or refute the use of H1 antihistamines alone in the management of eczema.

In an earlier review, in which the authors aimed to summarize the evidence for the efficacy of oral H1 antihistamines in relieving itch in people with eczema, it was concluded that most studies were underpowered, suffered from flawed study designs and that there was little objective evidence for the efficacy of antihistamines for itch relief in eczema patients [10]. A similar conclusion was reached in a more recent review [11]. However, although we are in broad agreement with the conclusions drawn by the authors of both reviews, they failed to address the potential confounding effect of the additional concomitant treatments.

A further systematic review is planned, which will include studies allowing the use of concomitant therapies. This might help fill the gaps in the evidence, in particular by demonstrating the benefit or otherwise of oral H1 antihistamines used as add-on therapy.

## Conclusion

The results of this systematic review demonstrate that, despite the fact that clinicians continue to prescribe oral H1 antihistamines to patients with eczema, there is no reliable high-level evidence to support this clinical decision. No randomised trials comparing an oral H1 antihistamine with placebo or control were found, and although there are circumstances in health care where randomised trials are unnecessary because the effects of an intervention are so marked, this is not the case for oral H1 antihistamines. We realise that the original, but apparently untested, idea behind the use of this class of drugs in eczema might have been their sedative effect, but even this rationale is surely now undermined by the use of newer drugs that cause little or no sedation. It would appear that oral H1 antihistamines bypassed what should be the key first step before the introduction of a drug for the treatment of a condition as common as eczema, namely research to prove the concept that the drug is better than nothing.

## Endnotes

This paper is based on a Cochrane review published in Issue 2, February 2013 of The Cochrane Library (see http://www.CochraneLibrary.net for further information) [4]. Cochrane reviews are regularly updated as new evidence emerges and in response to comments and criticisms. The Cochrane Library should be consulted for the most recent version of this review.

## Abbreviations

RCT: randomised controlled trial.

## Competing interests

The authors declare that they have no competing interests.

## Authors’ contributions

EJvZ led the preparation of the present manuscript. CJA led the systematic review. CJA, EJvZ, ZF and AJ independently assessed the titles and abstracts for eligible RCTs. EJvZ, UM and EW searched for ongoing and unpublished trials and screened conference proceedings. All authors participated in the systematic review and reviewed the draft versions of the manuscript. All authors read and approved the final manuscript.
